# The Role of Gammaherpesviruses in Cancer Pathogenesis

**DOI:** 10.3390/pathogens5010018

**Published:** 2016-02-06

**Authors:** Hem Chandra Jha, Shuvomoy Banerjee, Erle S. Robertson

**Affiliations:** Department of Microbiology and Tumor Virology Program, Abramson Comprehensive Cancer Center, Perelman School of Medicine at the University of Pennsylvania, 201E Johnson Pavilion, 3610, Hamilton Walk, Philadelphia, PA 19104, USA; hemjha@upenn.edu (H.C.J.); shuvogene@gmail.com (S.B.)

**Keywords:** EBV, KSHV, cancer, pathogenesis, epigenetics, apoptosis, autophagy, cell cycle, LANA, EBNA3C

## Abstract

Worldwide, one fifth of cancers in the population are associated with viral infections. Among them, gammaherpesvirus, specifically HHV4 (EBV) and HHV8 (KSHV), are two oncogenic viral agents associated with a large number of human malignancies. In this review, we summarize the current understanding of the molecular mechanisms related to EBV and KSHV infection and their ability to induce cellular transformation. We describe their strategies for manipulating major cellular systems through the utilization of cell cycle, apoptosis, immune modulation, epigenetic modification, and altered signal transduction pathways, including NF-kB, Notch, Wnt, MAPK, TLR, *etc.* We also discuss the important EBV latent antigens, namely EBNA1, EBNA2, EBNA3’s and LMP’s, which are important for targeting these major cellular pathways. KSHV infection progresses through the engagement of the activities of the major latent proteins LANA, v-FLIP and v-Cyclin, and the lytic replication and transcription activator (RTA). This review is a current, comprehensive approach that describes an in-depth understanding of gammaherpes viral encoded gene manipulation of the host system through targeting important biological processes in viral-associated cancers.

## 1. Introduction

### 1.1. Relationship between Viruses and Cancers

Information collected over the past 50 years has made it remarkably apparent that several viruses play major roles in the multistage process related to development of human malignancies. It is estimated that viral infections contribute to 15%–20% of all human cancers [1]. Oncogenic viruses have developed multiple intricate strategies to deregulate important biological functions of infected host cells. During cell division, the genetic materials of these pathogenic viruses can replicate in concert with the host cell division, while sustaining minimal exposure to the immune system as well as inhibiting apoptosis; these viruses can prevent cells from self-destruction [2]. Frequently, these viruses increase telomerase activity to push infected cells towards immortality [3,4]. Additionally, virus-infected cells can spread to other sites of the body, thus facilitating further proliferation and transmission of virus particles by altering cell-to-cell adhesion properties [5]. Thus, oncogenic viruses can be important experimental models for the investigation of multi-faceted cellular networks. These include regulation of tumor suppressors, identification of major signal transduction pathways for the maintenance of genomic integrity, and processes which govern cellular immune surveillance. Importantly, identification of cancer-associated viruses and their pathogenic roles with state-of-the-art technology will open new avenues for potential anti-viral therapies and vaccine strategies to combat human neoplasia.

Human tumor viruses belong to two virus families, the RNA virus families (e.g., Retroviridae and Flaviviridae) and the DNA virus families (e.g., Hepadnaviridae, Herpesviridae, and Papillomaviridae). Viruses associated with different types of human malignancies include, HTLV-1 (adult T-cell leukemia (ATL), HPV (cervical cancer, skin cancer, head and neck cancers, and anogenital cancers), Human herpesvirus-8 or HHV-8 (Kaposi’s sarcoma, primary effusion lymphoma, and Multicentric Castleman’s disease), Human herpesvirus-4 or EBV (Burkitt’s lymphoma, nasopharyngeal carcinoma, post-transplant lymphomas, and Hodgkin’s disease), and HBV and HCV (hepatocellular carcinoma). There are other viruses which can potentially contribute to human cancers including simian vacuolating virus 40 (brain cancer, cancer, and mesothelioma), BK virus (prostate cancer), JC virus (brain cancer), human endogenous retroviruses (germ cell tumors, breast cancer, ovarian cancer, and melanoma), human mammary tumor virus (breast cancer), and Torque teno virus (gastrointestinal cancer, lung cancer, breast cancer, and myeloma) [1].

### 1.2. Herpesviruses

Herpesviruses are known as large double-stranded DNA viruses with a genome size of 100 to 200 kilobases and are prevalent throughout the animal kingdom [6]. In humans, eight herpesviruses have been identified including: herpes simplex virus 1 and 2 (HSV-1 and HSV-2 or human herpesvirus (HHV)-1 and (HHV-2); varicella–zoster virus (VZV or HHV-3); Epstein–Barr virus (EBV or HHV-4); human cytomegalovirus (HCMV or HHV-5); human herpesviruses 6 and 7 (HHV-6 and HHV-7); and Kaposi’s sarcoma-associated herpesvirus (KSHV or HHV-8) [6]. Interestingly, all herpesviruses share a common evolutionary origin, as evidenced by the higher amino acid sequence similarity between different herpesviral proteins [6]. Herpesviruses maintain their genomes as extrachromosomal circular episomes in the nuclei of infected cells without the need for integration. However, several reports of chromosomally integrated herpesvirus (CIHHV) suggested that herpesviruses can indeed integrate into the host’s chromosomes under certain conditions. In addition, human herpesvirus 6 (HHV-6) was found to be integrated into the germ lines of approximately 1% of the world’s population, and integration may represent more than a sporadic or anecdotal event [7]. They are often referred to as co-carcinogens [8]. Herpesviruses have been found to also be etiologic agents in some chicken, primate, and frog neoplasms and potentially contribute to lymphomagenesis in human [9].

Herpesviruses are divided into three subfamilies: α, β and γ. This classification of herpesviruses is mainly based on their genome sequence, organization, and biological features [6]. The γ-subfamily of herpesviruses is lymphotropic, and some are capable of undergoing lytic replication in epithelial cells [10]. These viruses establish a lifelong period of latency in host cells, with intermittent periods of lytic replication. The γ-herpesviruses show relatively similar genome organization and share more genes with each other than with members of either the α- or β-subfamilies of herpesviruses [6].

### 1.3. Gammaherpesviruses

**Figure 1:**
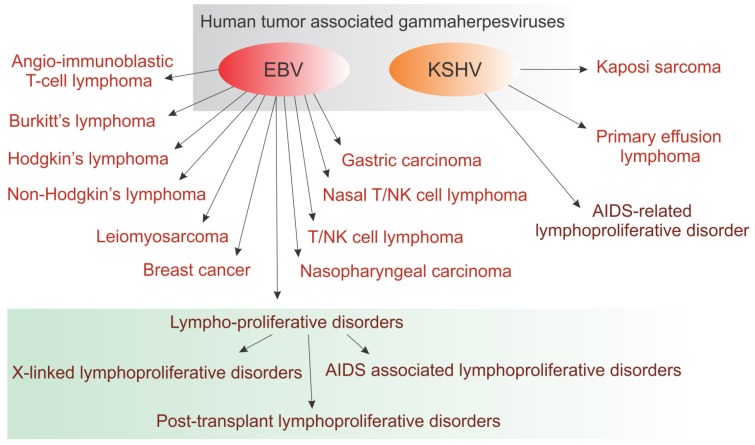
Human tumor associated gammaherpesviruses EBV and KSHV are related to a wide range of human malignancies.

The gammaherpesviruses can be divided into two genera: lymphocryptoviruses and rhadinoviruses. Lymphocryptoviruses have been identified in higher primates, whereas rhadinoviruses have been identified in a wide range of mammalian species. Two types of gammaherpesviruses have been identified in humans: Epstein-Barr virus (EBV; genera-lymphocryptovirus), and Kaposi’s sarcoma–associated herpesvirus (KSHV; genera-rhadinovirus). The human gammaherpesviruses are able to establish a lifelong, persistent infection in immunocompetent hosts [11]. However, the gammaherpesviruses are responsible for a variety of lymphoproliferative and neoplastic disorders. For example, HHV-8 or Kaposi’s sarcoma-associated herpesvirus-associated neoplasia, which includes KS (Kaposi’s sarcoma), MCD (Multicentric Castleman’s disease) and PEL (primary effusion lymphoma) (Figure 1) [12]. Additionally, HHV-4 or EBV, is etiologically associated with infectious mononucleosis, Burkitt’s lymphoma, nasopharyngeal carcinoma (NPC), Hodgkin’s disease, hemophagocytic lymphohistiocytosis syndrome and some gastric cancers (Figure 1) [11]. EBV and Kaposi’s sarcoma-associated herpesvirus (KSHV) association with a wide range of human malignancies is schematically represented in Figure 1. Herpesvirus Saimiri (HVS) belongs to Rhadinovirus genera and is an example of a non-pathogenic virus in its natural host, the squirrel monkey, and establishes lifelong persistence, primarily in T-lymphocytes [13]. Interestingly, cross-species transmission of HVS was found in other new world primates, such as the common marmoset and thereby can induce hematological malignancies like, Lymphoma, lymphosarcoma, and leukemia [14]. Notably, the primate γ-herpesvirus HVS is shown to be lymphotropic and capable of inducing T-cell neoplasia [6].

### 1.4. Human Tumor-Associated Gammaherpesviruses

#### Role of Epstein-Barr Virus (EBV) in Human Malignancies

EBV was the first gammaherpesvirus discovered in 1964 by Epstein, Achong and Barr. EBV was also found in all cases of the endemic form of Burkitt’s lymphoma (BL) [15]. Shortly, after its discovery, Henle and his coworkers reported that EBV infection of umbilical cord lymphocytes resulted in the transformation of B-cells to continuously proliferating lymphoblastoid cell lines (LCLs) [16]. This valuable observation suggested a clear connection between EBV infection and development of lymphoma. Extensive research in the field of viral oncogenesis, utilizing LCLs, as well as other cell lines established from BL tumors, identified different viral antigens expressed in these cells and further determined which of these gene products are essential for primary human B-cells immortalization [17]. Primarily, EBV infection is transmitted by saliva and occurs in childhood in an asymptomatic manner. EBV has the capacity to actively replicate in the epithelial cells of the oropharynx and has subsequent potential to infect circulating B-lymphocytes. Up to 50% of adolescent’s primary EBV infection may lead to a non-malignant symptom, known as acute infectious mononucleosis [18]. This specific symptomatic condition is the consequence of the powerful antiviral T cell response to the EBV-driven B-cell proliferation [19]. EBV exerts its potential oncogenic property by maintaining its viral genome in the cell, avoiding the killing of the cell, and preventing the cell from becoming a target for destruction by the immune system. EBV latent genes induce an activated phenotype in infected B-cells. Although these cells are not transformed, they proceed unimpeded, acquire oncogenic mutations, and can become neoplastic [20].

### 1.5. EBV Associated Cancers

#### 1.5.1. Burkitt’s Lymphoma

Burkitt’s lymphoma (BL) is an aggressive B-cell malignancy which is classified into three forms referred to as endemic (eBL), sporadic (sBL) and HIV-associated BL, depending upon geographic distribution and EBV-association [21]. The “endemic” or high-incidence form of BL occurs at an annual incidence rate of approximately 5–10 cases per 100,000 children living in the equatorial belt of Africa and in some parts of Papua New Guinea [22]. In contrast, worldwide sporadic cases of BL occur at a lower frequency. Whereas virtually every BL tumor found in high-incidence regions is EBV positive, only 15% of sporadic BL are positive for EBV. Additionally, some “intermediate-incidence” areas outside the regions of holoendemic malaria, such as Algeria and Egypt, have increased the number of cases that correlates with an increased proportion of EBV-positive tumors. BL incidence is also observed as a consequence of HIV infection. The precise role of EBV in BL pathogenesis remains to be elucidated, although the detection of monoclonal EBV episomes in virus-positive BL biopsies suggests a possibility that EBV infection preceded proliferation of the precursor B-cells [23]. The occurrence of BL in the germinal center is based on phenotypic studies and is supported by the ability of other BL risk factors, such as holoendemic malaria and chronic HIV infection, which stimulate the proliferation of B-cells in the germinal center [24]. Interestingly, BL cells from primary tissues show a very restricted viral gene expression pattern, where EBNA1 was found to be the only EBV antigen consistently detected [25]. In addition, some other reports have suggested the expression of LMP1 and EBNA2 in a few cases of endemic BL [26]. In culture condition, EBV-positive BL cells can express other EBV nuclear antigens as well as latent membrane proteins, and the EBNA2- and LMP1-induced cell surface antigens, such as CD23, CD30, CD39, LFA1, LFA3 and ICAM1, are upregulated [25]. Upon EBV infection, deregulated MYC expression was observed as it helps in EBV-driven cell proliferation. These findings may enable the infected cells to evade Cytotoxic T Lymphocyte (CTL)-mediated immune surveillance [24]. Introduction of activated MYC gene into an EBV-transformed cell line in which EBNA2 was rendered null was shown to be capable of inducing continuous proliferation of these cells in the absence of functional LMP1 and EBNA2. This suggests the possibility that MYC can substitute for LMP1 and EBNA2 in BL progenitor cells [27]. Thus, rearranged defective EBV genomes have been detected in some sporadic BL tumors [28].

#### 1.5.2. Hodgkin’s Disease

In 1966, MacMahon proposed that Hodgkin’s disease might be caused by an infectious agent [29]. The first evidence that it is linked to EBV infection was provided by the detection of raised antibody titers to EBV antigens in patients with Hodgkin’s disease when compared with patients with other lymphomas [30]. An earlier report suggested that EBV had a direct or indirect role in the pathogenesis of Hodgkin’s disease, either by triggering several pathogenic mechanisms or by modulating the process of immuno-regulation which supported the malignancy and reactivation of the virus [31]. Interestingly, EBV DNA was detected primarily in 20%–25% of Hodgkin’s disease tumors by Southern blot hybridization techniques with cloned viral probes [32]. The existence of EBV DNA in HRS (Hodgkin-Reed Sternberg) cells was first detected by using *in situ* hybridization [33]. Consequently, other studies have identified abundant EBV early RNA (EBER1 and EBER2) sequences in HRS cells which is important for detecting latent infection *in situ* [34]. Interestingly, EBV positive Hodgkin’s disease appear to be less common in the population of developed countries, with percentages of between 20% and 50% for North American and European cases, respectively [35], and 57%–59% for Hodgkin’s disease in China [36], but it shows much higher rates in underdeveloped countries such as Peru [37] and Kenya [38]. In underdeveloped countries, the growing incidence of EBV positive Hodgkin’s disease might be a consequence of an existing underlying immunosuppression similar to African Burkitt’s lymphoma in malaria-infected populations [37].

#### 1.5.3. Non-Hogkin’s Lymphoma in Immune Competent Populations

EBV has the potential to infect different immune cells [20]. Non-Hodgkin’s lymphomas (NHLs) are associated with EBV infection [39]. EBV is strongly linked with Nasal T/natural killer non-Hodgkin’s lymphomas, irrespective of geographical location [40]. Angioimmunoblastic lymphadenopathy, an atypical T-cell lymphoma in which expanding B-cell clones are often present beside the T-cell clones [40]. EBV infection is mainly seen in B-lymphocytes and B-immunoblasts, although the virus occurs in rare neoplastic and non-neoplastic T-cells [41]. EBV-positive B-cells have also been observed in growing peripheral T-cell lymphomas [42]. These findings also suggest possible activation of EBV in latently infected B-cells by the neoplastic T-cells, and a role for EBV-positive B-cells in maintaining the malignant T-cell process [43].

#### 1.5.4. Nasopharyngeal Carcinoma

The association of EBV with undifferentiated nasopharyngeal carcinoma is well characterized, whereas the association with the other two subtypes of nasopharyngeal cancers is still a matter of controversy [44]. Undifferentiated nasopharyngeal carcinoma affects mostly middle-aged individuals and is more common in men [44]. Almost every undifferentiated nasopharyngeal carcinoma was found to be EBV positive, regardless of geographical origin [20]. In undifferentiated nasopharyngeal carcinoma, EBV infects the epithelial cells of the posterior nasopharyngeal region in Rosenmuller’s fossa of the Waldeyer’s ring [45]. Interestingly, two models have been postulated to explain the mode of EBV infection in these cells. Firstly, the CD21 receptor has been described as one of the points of entry by EBV [46]. Alternatively, it was suggested that EBV may enter into nasopharyngeal cells through IgA-mediated endocytosis pathways [47]. Existence of EBV was also detected in nasopharyngeal carcinoma *in situ*, a known precursor of undifferentiated nasopharyngeal carcinoma [48]. These findings suggest the potential role of EBV in the progression of this malignant phenotype.

During the 1980s there were two distinct types of EBV identified, referred to here as EBV1 and EBV2 [49]. Both EBV types are widely distributed in human populations and appear to have near equivalent biological properties, although lymphocytes transformed to an indefinite growth phenotype by EBV1 propagate more vigorously than cells transformed by EBV2 [50]. Interestingly, EBV-1 and EBV-2 have both been implicated in nasopharyngeal carcinoma. The majority of nasopharyngeal carcinoma cases from individuals in Southern China, Southeast Asia, Mediterranean, Africa, and the United States are associated with EBV-1 infection [51]. In contrast, some cases involving Alaskan Inuits are mostly EBV-2-related but contain polymorphisms with characteristics of Asian EBV-1 [51]. EBV shows latency II expression in undifferentiated NPC [48]. Additionally, a cytogenetic abnormality was found to be associated with undifferentiated nasopharyngeal carcinoma, often described as non-random deletion in short arm of chromosome 3 at loci 3p25 and 3p14 [52]. Interestingly, NPC cells possess normal antigen processing and they are effectively recognized by EBV-specific CTLs, yet they are not destroyed [53]. It has been reported that EBV-encoded viral IL-10 is increased in NPC and has been associated with increased production of IL-1 and also produced by epithelial cells and by CD4^+^ T cells, which may, in turn, contribute to the growth of the tumor and immune evasion [54]. Furthermore, over-expression of Bcl-2 may also play a role in progression of this malignant disease by allowing the cell to bypass apoptosis [55].

#### 1.5.5. T/NK-Cell Lymphoma

EBV primarily infects B-cells, although it has the potential to infect other cells. Interestingly, several types of non-B-cell NHL are associated with EBV [41]. It has been reported that different T-cell lymphoproliferative disorders are linked with EBV infection including a subset of peripheral T-cell lymphomas, angioimmunoblastic T-cell lymphoma (AILT), extranodal nasal type NK/T-cell lymphoma, enteropathy type T-cell lymphoma, hepatosplenic and nonhepatosplenic T-cell lymphomas, EBV-associated cutaneous T-cell lymphoproliferative disorders and aggressive NK-cell leukemia or lymphomas [56].

#### 1.5.6. Angioimmunoblastic T-cell Lymphoma

Angioimmunoblastic T-cell lymphoma (AILT) is a type of Peripheral T-cell lymphomas (PTCL) and characterized by systemic disease, a polymorphous infiltrate primarily involving lymph nodes, endothelial venules and follicular dendritic cells [57]. AILT is well-known as the second most common PTCL subtype, accounting for 15%–20% of cases [57]. EBV infection is seen mainly in the B-lymphocytes and B-immunoblasts, although virus infection occurs in rare neoplastic and non-neoplastic T cells. Actually, the presence of EBV in only a sub-population of cells suggests that EBV infection is secondary to malignancy or that the viral genome has been lost from the malignant cell [20].

#### 1.5.7. Nasal T/NK-Cell Lymphoma

Nasal T/NK-cell lymphoma cells show several unique genotypic and phenotypic features. The characteristic features include an absence of T-cell antigens, the expression of the NK cell marker CD56, and the absence of T-cell receptor gene rearrangement [58]. Interestingly, EBV is found consistently associated with these lymphomas, regardless of their geographical distribution [59].

#### 1.5.8. Gastric Carcinoma

Earlier reports suggested that the presence of EBV varies from 90% in lympho-epithelioma like gastric carcinomas to between 5 and 25% in gastric adenocarcinomas [60]. But the pathogenic role of EBV in either of these two tumors is still not clear [61]. It has been reported that, in lymphoepithelioma-like gastric carcinoma, EBV spreads from the nasopharynx to the stomach [62]. In regard to gastric adenocarcinomas, EBV may enter the gastric epithelial cells without the use of a receptor. Detailed clinical investigations suggested that this is accomplished by the binding of IgA antibody with EBV particles derived from B-lymphocytes and the uptake of these particles by gastric epithelial cells [63]. Alternatively, EBV may enter the gastric epithelial cells via a receptor other than the CD21 receptor [64]. Additionally, EBV shows a novel latency pattern in gastric adenocarcinomas that includes the production of BARF-1, a homologue to human colony-stimulating factor 1 receptor and intracellular adhesion molecule 1, and the absence of LMP-1 [65]. It has been demonstrated by several studies that there is a delay in apoptosis in EBV-positive gastric carcinomas (up-regulation of BCL-2 and p53) and a decrease in cellular differentiation (down-regulation of E-cadherin) [66].

#### 1.5.9. Breast Cancer

The relationship between EBV and breast cancer is controversial. Some studies have reported an EBV incidence in breast cancer tissue about 21%–51% [67], whereas other clinical investigations have failed to detect EBV in any breast cancer tissue samples [68]. There are possibilities for these discrepancies which include (1) distinct EBV detection techniques; (2) differing EBV-derived proteins or RNAs analyzed; (3) epidemiological variation in EBV infections or in breast cancer itself. Regardless, whether EBV is present in breast cancer, and its possible etiological role in oncogenesis, remains to be elucidated.

#### 1.5.10. Leiomyosarcoma

Leiomyosarcomas are considered smooth muscle tumors. Although, they are not associated with EBV in immune competent hosts they have a strong correlation with viral infection in patients whose immune system is compromised by HIV or other factors [69]. These observations also indicate that EBV is capable of infecting smooth muscle cells, a finding consistent with experimental evidence that the EBV receptor is present on those cells [69].

### 1.6. Role of Kaposi’s Sarcoma Associated Herpesvirus (KSHV) in Human Malignancies

In 1994, KSHV or human herpesvirus 8 (HHV8) was discovered in AIDS-associated KS (Kaposi’s Sarcoma) lesions [70]. Interestingly, the presence of KSHV was reported in all cases of KS that arise in HIV-infected and HIV-negative individuals. Additionally, KSHV was found to be associated with the development of a rare B-cell lymphoma called primary effusion lymphoma (PEL) and multicentric Castleman’s disease (MCD), which is a typical lymphoproliferative disorder characterized by the expanded germinal centers with B-cell proliferation and vascular proliferation [71]. KSHV-positive MCD is now recognized as a distinct subset of MCD, called plasmablastic MCD, which contains large plasmablastic cells [72]. Dysregulated IL-6 levels are considered a probable contributor to the clinical pathophysiology of MCD [73]. Like KS and PEL, KSHV genomes are detectable in almost all HIV-seropositive MCD cases and approximately 50% HIV-seronegative MCD cases [74].

#### 1.6.1. Kaposi’s Sarcoma (KS)

KS is designated as an unusual multifocal neoplasm characterized by dark purple lesions, which differs from most other common tumors in that the lesions contain multiple cell types [75]. KS lesions contain specific features of extensive neo-angiogenesis, infiltrating inflammatory cells, erythrocyte extravasation, endothelial cells, and characteristic “spindle” cells, which are typical for KS. Four distinct clinical variants of KS were defined which are based on the extent of immunosuppression and severity of infection. They are categorized as classic KS, endemic KS, iatrogenic KS, and AIDS-associated KS [76]. Classic KS most commonly presents in HIV-negative elderly male patients of Mediterranean and Eastern European decent and is relatively indolent [77]. Endemic KS is the prevalent form in Equatorial, Eastern, and Southern Africa, and is substantially more aggressive than classic KS [78]. Unlike classic KS, endemic KS often presents in HIV-negative children as a lymphadenopathy [79]. Iatrogenic or post-transplant KS is developed in patients undergoing immunosuppressive therapy to prevent graft rejection after the organ transplantation [80]. 

#### 1.6.2. Primary Effusion Lymphoma (PEL)

PEL is also referred to as body cavity-based lymphoma (BCBL). It is a rare, rapidly fatal lymphoma related to KSHV infection and commonly found in HIV-infected patients [81]. PEL is a unique form of NHL found more commonly in immunocompromised AIDS patients, and, unlike KS, PEL is generally presented as a pleural or pericardial effusion without a detectable tumor mass [82]. It can also be presented as a solid mass in the lymph nodes, lungs, or the gastrointestinal tract [83]. Due to the presence of hyper mutated immunoglobulin genes and markers of late stage B-cell differentiation such as CD30 and CD138, PEL cells are thought to be usually monoclonal and originated from post-germinal center B-cells [84]. In PEL cells, it has frequently been seen that KSHV presents as a single positive or KSHV/EBV double-positive, and that the KSHV genome is maintained at a relatively high copy number (50–150 per cell) [85].

### 1.7. Lymphoproliferative Disorders in Immunocompromised Populations Induced by Human Gammaherpesvirus Infection

#### 1.7.1. Role of EBV

Immuno-compromised individuals present a lack of T-cell control which ultimately favors the expansion of B-cell clones that are infected and immortalized. These cells may also gain additional genetic lesions which causes oligoclonality and, ultimately, monoclonality of the B-cell proliferation [86]. The status of immune-compromised patients after hemopoietic stem cell transplantation or solid organ transplantation may destroy the normal balance between proliferative capacity of latently infected B-cell and also the EBV-specific T-cell response. Therefore, the increased number of latently infected B-cells may lead to aggressive post-transplant lymphoproliferative disorders (PTLD) [87]. Firstly, X-linked lymphoproliferative disorder results from an inherited immunodeficiency, and, secondly, there are lymphomas that develop due to immunosuppressive drugs given to the transplant recipients. Additionally, there are AIDS-related lymphoproliferative disorders. Interestingly, the most common gene expression pattern in these disorders was found to be EBV latency III.

#### 1.7.2. X-Linked Lymphoproliferative Disease

X-Linked lymphoproliferative disease is characterized by fatal or fulminant infectious mononucleosis, B-cell lymphomas, and dysgammaglobulinemia, and most of the lymphomas are reported as extra-nodal, and they often involve the intestine [88]. EBV infection was considered a major trigger for immune function deregulation. The responsible gene for this disorder has been mapped to the long arm of the X chromosome (Xq24), and it is designated as SH2D1A/SAP [89]. It was reported that defects in this gene may lead to reduced ability to regulate immune responses against viruses, including EBV [90].

#### 1.7.3. Post-Transplant Lymphoproliferative Disorders

These disorders mainly arise in the situation of therapeutic immunosuppression after organ transplantation [91]. Nearly all forms of these aggressive disorders have EBV association, and EBV infected polyclonal B-cell populations are more susceptible to genetic variations with altered BCL-6 gene expression [92]. Subsequently, emergence of other molecular aberrations finally drives malignant growth [92]. The incidence of post-transplant lymphoproliferative disorders varies greatly depending on the types of organ being transplanted, the status of EBV in the transplant recipient and donor, and the therapeutic strategies used to achieve immunosuppression [93]. Commonly, the disorder occurs in combined liver-kidney transplants cases, followed by cardiac, liver, lung, and then kidney transplants. A variety of distinct post-transplant lymphoproliferative disorders have been defined, including plasmacytic hyperplasia, polymorphic lymphoproliferative disorder, malignant non-Hodgkin’s lymphoma, and multiple myeloma [94]. Most of the post-transplant-lymphoproliferative disorders (PTLDs) are considered B-cell malignancies. Molecular detection is very essential for the diagnosing and monitoring of patients who are affected by these diseases [95]. Immuno-staining of EBV-LMP-1 and EBER hybridization is used as routine detection technique for identifying latent EBV in PTLD affected tissues.

### 1.8. Aids-Related Lymphoproliferative Disorders

#### 1.8.1. Role of EBV

AIDS-related lymphoproliferative disorders are a heterogeneous group of diseases which mainly arise in the presence of HIV-associated immunosuppression, a condition that permits the uncontrolled proliferation of EBV-infected lymphocytes. Moreover, pleural effusion lymphomas also occur and often show the existence of EBV in addition to human herpesvirus 8. EBV association is frequently found in AIDS-related central nervous system lymphomas which are derived from germinal center B-cells [91]. The central nervous system lymphomas are classified into two types: immunoblastic and large non-cleaved lymphomas. Generally, the immunoblastic subtype expresses LMP-1 and BCL-2, but not BCL-6. Interestingly, the large non-cleaved subtype expresses BCL-6, but not LMP-1 or BCL-2 [96]. The AIDS-related systemic lymphomas are included in several subtypes, such as diffuse large cell lymphomas, immunoblastic lymphomas, Burkitt’s lymphomas, and small, and non-cleaved Burkitt’s-like lymphomas. EBV positivity was found in 30% to 90% of these lymphomas [97].

#### 1.8.2. Role of KSHV

Primary effusion lymphomas (PELs) are rare tumors and characterized by pleural, pericardial or peritoneal lymphomatous effusions in the absence of a solid tumor mass [98]. Basically, KSHV targets both endothelial cells and B-lymphocytes and suggests a possible interaction between PEL cells and endothelial cells [99]. The majority of PELs occurring in HIV-infected individuals are latently infected with both EBV and KSHV. Recent studies support the idea that KSHV plays a transforming role in PELs, and that expression of a variety of genes is crucial for proliferation and survival of infected tumor cells [100]. Generally, three viral gene products are clearly expressed in all latently infected cells from a single promoter in a tri-cistronic transcript, such as- LANA, vCYC and vFLIP. However, other viral gene products have also been shown to be expressed in different lymphoproliferative disorders [100]. In particular, PEL cells contain multiple copies of episomal KSHV genomes [101]. Mostly, viral gene expression pattern involves expression of LANA, a viral D-type cyclin homologue (vCyc), a viral homologue of FLICE inhibitory protein (vFLIP), a pre-miRNA transcript encoding 11 viral miRNAs, as well as vIRF3. In addition, a homologue of IL-6 (vIL-6) is also expressed in some PEL cells [102]. 

## 2. Role of Major EBV Antigens in Cancer

### 2.1. EBNA1

EBV-encoded multifunctional latent protein Epstein-Barr Nuclear Antigen 1 (EBNA1) is directly involved in progression of carcinogenesis. Previous reports suggested that B-cell-directed EBNA1 expression can lead to production of B-cell lymphomas in transgenic mice [103]. EBNA1 is expressed in all forms of latency [104], and is linked with some EBV-associated tumors by inducing genomic instability [105]. EBNA1 was also shown to be important for efficient EBV-mediated immortalization of B-cells *in vitro* [106]. It is a homo-dimeric, DNA-binding protein that binds site-specifically to its cognate DNA sequence [107] via its DNA-binding and dimerization domain located at its C-terminal end. EBNA1 facilitates viral genome synthesis by binding to clusters of EBNA1-binding sites within the EBV latent origin of replication (OriP) [108], and the nonrandom partitioning of newly synthesized viral plasmids to daughter cells during mitosis [109]. Earlier reports demonstrated that EBNA1 plays a major role in the continued proliferation or survival of EBV-positive tumor cells. Enhanced expression of EBNA1 mutants which have a dominant-negative effect on EBNA1 function was found to decrease cell survival and increase the rate of apoptosis in EBV-positive Burkitt’s lymphoma cells but not in EBV-negative B-cells [110]. Furthermore, inhibition of EBNA1 in Burkitt’s lymphoma or NPC cell lines reduced cellular proliferation [111]. These experiments designate an important contribution of EBNA1 for B-cell immortalization but are still not clear whether EBNA1 contributed directly to cell proliferation and survival or was simply required to maintain the EBV genome as well as expression of other EBV oncoproteins. Evidence from *in vivo* studies has suggested that EBNA1 expression without EBV infection may be adequate to promote cell proliferation and survival for tumor induction. Wilson *et al.* has shown that EBNA1 can directly affect cell transformation [103]. However, the role of EBNA1 in cell transformation is still not fully understood. Interestingly, EBNA1 expression was found to be a major contributor to enhanced tumor progression in nude mice model [112]. Additionally, EBNA1 expression in a breast carcinoma cell line promoted the rate of tumor growth in nude mice as well as increased lung metastases [113]. Our Lab also showed that EBNA1 interaction with the metastasis suppressor protein Nm23-H1 in lymphoblastoid cell lines inhibited its ability to suppress cell migration [114]. These studies strongly suggested a potential role for EBNA1 in altering cellular properties to promote carcinogenesis. 

### 2.2. EBNA2

The role of EBNA2 in growth transformation was first revealed with studies in EBV infected Burkitt’s lymphoma cell line, P3HR-1. P3HR-1, a mutant form of EBV, is non-transforming due to a deletion that removes all the EBNA2 coding regions and the last two exons of EBNA-LP [115]. Interestingly, introduction of DNA fragments spanning the EBNA2 reading frame by homologous recombination restored the transformation capacity of the virus. Therefore, EBNA2 was considered to be essential for primary B-cell growth transformation [17]. Additionally, other experiments have shown that EBNA2 is not only important for the initiation but also for the maintenance of B-cell immortalization [116]. As a transcriptional activator of both cellular and viral genes, EBNA2 upregulates the expression of CD21 and CD23 as well as LMP1 and LMP2 [117]. Ubiquitous DNA-binding protein, RBP-Jκ was found to interact with EBNA2 and this is partially responsible for targeting EBNA2 to promoters containing RBP-Jκ cognate sequence [118]. Other reports suggested that EBNA2 has the capacity to functionally replace the intracellular region of Notch [119], and is important for EBV-induced B-cell proliferation by targeting transcription of the c-myc oncogene [120].

### 2.3. EBNA3A

EBNA3A and EBNA3C were reported as essential latent antigens for the generation of LCLs, whereas EBNA3B was found dispensable [121]. The EBNA3 antigens EBNA3A, EBNA3B, and EBNA3C are stably associated with RBP-Jκ [122]. Interestingly, three- to five-fold enhancement of EBNA3A expression in LCL interrupts the association between EBNA2 with RBP-Jκ and downregulates c-Myc, CD21, and CD23, and also causes G_0_/G_1_ arrest [123]. It was also observed that EBNA3A co-activates the LMP1 promoter in association with EBNA2 [124]. EBNA3A, EBNA3B, and EBNA3C all possess common and evolutionarily conserved domains [125]. EBNA3A and EBNA3C both interact with a wide range of cellular proteins which may mediate transcriptional activation, repression or affect cell proliferation [126].

### 2.4. EBNA3B

In contrast with other members of EBNA3, EBNA3B was observed to be dispensable for B-cell transformation *in vitro*. Interestingly, EBNA3B has not been counter-selected over millions of years of virus-host co-evolution, which again suggests its important role *in vivo* [127]. EBNA-3B was first identified as a 165-kDa nuclear protein, and in parallel studies found that multiple BamHI-E DNA fragments transfected into Cos-1 cells, yielding EBNA-3B [128]. Another report suggested that the EBNA-3 family of proteins including EBNA-3B are primary antigenic targets for cytotoxic T-cell responses against immortalized B-cells [20].

### 2.5. EBNA3C

**Figure 2:**
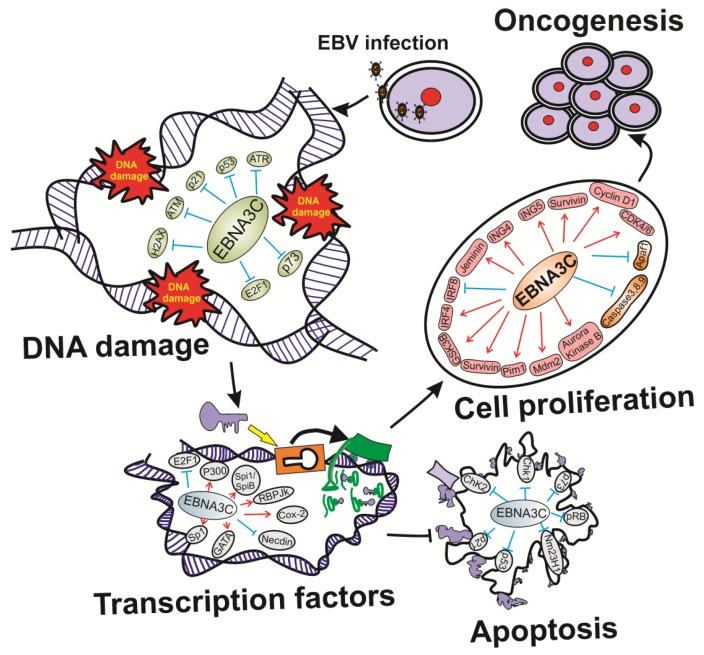
Diagram represents the multi-faceted activities of EBV latent antigen EBNA3C in regulation of DNA damage response, host transcription factors, cell cycle regulatory proteins, cell proliferation and apoptosis during EBV infection.

One of the essential EBV latent antigens EBNA3C is necessary for growth transformation of primary B-lymphocytes *in vitro* and regulates the transcription of a number of viral and cellular genes important for the immortalization process (Figure 2) [128]. EBNA3C performed as a potent transcriptional co-regulator that interacts with a wide range of cellular proteins which are essentially involved in modulating several important cellular processes, including cell cycle regulation and apoptosis (Figure 2) [129,130,131]. Our lab demonstrated the role of EBNA3C to form stable complexes with different transcriptional co-repressors as well as chromatin modification enzymes (Figure 2) [132,133]. Several studies showed that EBNA3C may recruit HDAC activities by transcriptional repression, as trichostatin A, a known pan-HDAC inhibitor, impedes EBNA3C-mediated transcriptional repression from the major Cp latent promoter [134]. Consequently, our lab also demonstrated that EBNA3C can not only make stable complexes with various HDACs and HATs but also with other transcriptional co-factors, including mSin3A, Prothymosin-α, and NCoR in EBNA3C-expressing B-cells [132]. Previous reports suggested that EBNA3C has an important role in reversing Nm23-H1-mediated inhibition of cellular migration after co-expression with Nm23-H1 in a breast carcinoma cell line and an EBV-negative BL cell line, demonstrating that EBNA3C can potentially promote metastasis in EBV-positive tumors by modulating Nm23-H1 activities (Figure 2) [135]. Additionally, EBNA3C was found to regulate p53 functions by enhancing Mdm2-mediated p53 ubiquitination and degradation by deubiquitination of Mdm2 (Figure 2) [136], as well as by targeting the inhibitor of growth family proteins 4 and 5 (Figure 2) [135]. Moreover, EBNA3C was found to be crucial for interacting with several cellular kinases and deregulating their functions which ultimately contribute to B cell transformation (Figure 2) [137,138]. A detailed functional attribution of EBNA3C activities with host has been drawn in Figure 2.

### 2.6. LMP1

EBV latent membrane proteins 1 (LMP1) is frequently expressed in nasopharyngeal carcinoma (NPC), gastric cancer, Hodgkin lymphoma, Burkitt’s lymphoma, AIDS and post-transplant lymphomas and have profound effects on cellular signaling pathways and cellular growth *in vitro* [139]. Reports suggested that LMP1 has important roles for modulating several cellular processes including differentiation, migration, and tumorigenicity [140]. Studies with genetic deletion recombinant viruses have revealed that LMP1 is one of the latent genes required for *in vitro* B-cell immortalization by EBV [141]. Importantly, LMP1 also plays an oncogenic role in non-lymphoid cells, and studies suggested that it has the ability to induce growth transformation in certain immortalized rodent fibroblast cells [142]. Additionally, the expression of LMP1 in the epidermis of transgenic PyLMP1 mice induces early pre-carcinogenic hyperplasia [143]. Moreover, *in vitro* studies revealed that heterologous expression of LMP1 leads to reduced serum requirements, loss of anchorage dependence, increased invasive capacity, and inhibition of terminal differentiation in cultured carcinoma cell lines [139].

### 2.7. EBERs

EBERs are EBV-encoded small non-polyadenylated, noncoding (nc) RNAs, also referenced to as EBV-encoded small RNAs [144]. They are the most abundant viral transcripts in latently EBV-infected cells [145]. They are 167 and 172 nucleotides long and transcribed by RNA polymerase III (pol III) [146]. Due to their abundance, EBERs can be used as target molecules for detecting EBV-infected cells in tissues by in-situ hybridization [147]. Previous reports demonstrated the roles of EBERs in EBV-mediated oncogenesis. Importantly, EBERs were found to play a crucial role for maintaining the malignant phenotypes of Burkitt’s lymphoma cells [148]. Additionally, they confer resistance to protein kinase RNA-dependent (PKR)-mediated apoptosis in BL and epithelial cells [149]. Furthermore, EBERs induce transcription of cytokines including IL-10 in BL cells, insulin-like growth factor (IGF)-1 in epithelial cells, and IL-9 in T cells that act as an autocrine growth factor of those EBV-infected cancer cells [150]. Recent studies demonstrated that non-coding RNA (ncRNA) contributes to the pathogenesis of EBV infection through modulation of innate immune signals [151]. Comparison of EBV-positive and -negative cell clones revealed that the presence of EBV in Akata cells was required for the cells to be more malignant and resistant to apoptosis [152], which underlies the oncogenic role of EBV in progression of BL. Later studies revealed that EBERs were responsible for these phenotypes [148].

Studies by Kitagawa *et al.* demonstrated that EBERs induce human IL-10 expression in BL cells [153]. EBV associates with various T cell-proliferating diseases such as chronic active EBV infection and nasal lymphoma. A human T-cell line, MT-2, was susceptible to EBV infection, and EBV-infected cell clones showed type-II latency, which was identical with those seen in EBV-infected T cells *in vivo* [154]. It was found that EBV-positive MT-2 cells express higher levels of IL-9 than EBV-negative MT-2 cells at the transcriptional level, and EBERs were responsible for IL-9 expression [155]. It was also reported that 5%–10% of worldwide gastric carcinoma (GC) cases are associated with EBV [156]. Iwakiri *et al.* demonstrated that EBV infection induces expression of IGF-1 in the GC-derived EBV-negative cell line NU-GC-3, and the secreted IGF-1 acts as an autocrine growth factor [150]. Transfection of individual EBV latent gene into NU-GC-3 cells revealed that the EBERs were responsible for IGF-1 expression. These findings seem to be operative *in vivo*, as EBV-positive GC biopsies steadily express IGF-1, while EBV-negative GC biopsies do not [151]. Therefore, EBERs would directly affect the pathogenesis of EBV-positive GC. It was also reported that EBER induces IGF-1 expression in EBV-negative NPC-derived cell lines CNE1 and HONE1 [157].

## 3. Role of Major KSHV Antigens in Cancer

### 3.1. Lana

KSHV-encoded latency-associated nuclear antigen (LANA) is a latent protein regularly expressed in all KSHV-associated diseases. LANA is important for regulating viral as well as cellular gene expressions and also crucial for viral genome maintenance. LANA interacts with several tumor suppressor genes, such as-p53 and pRb, suggesting that LANA can play a role in promoting oncogenesis [158]. Studies by using fluorescent *in situ* hybridization proved that LANA associates with the KSHV genome during infection [159]. Additionally, experimental evidence showed that H2AX phosphorylation was essential for LANA-mediated KSHV episome persistence [160]. Binding of LANA with a number of cellular proteins involved in transcriptional regulation such as CBP, RING3, activating transcription factor-4/cyclic AMP response element binding protein-2 and mSin3A [161]. A recent study showed that LANA can induce chromosomal instability through targeted degradation of the mitotic checkpoint kinase Bub1 [162]. Additionally, LANA was found to be important for contributing to viral latent replication by activating phosphorylation of survivin [163]. Interestingly, IRF-4-mediated CIITA transcription was blocked by LANA to inhibit MHC II presentation in lymphoma cells [164]. In addition, LANA activates the EBV LMP1 promoter, suggesting a functional role in regulating EBV latent gene expression in co-infected BCBL1 cells, including the upregulation of the EBV oncogene, LMP1 [165].

### 3.2. v-Cyclin

KSHV-encoded v-cyclin is the viral homolog to cellular cyclin, most closely related to Cyclin D [166]. Cellular Cyclin D binds with cyclin dependent kinases (CDK6 and CDK4) [167], and this complex phosphorylates pRb for releasing the transcription factor E2F. Interestingly, v-Cyclin, like cellular cyclins, is also capable of phosphorylating pRb *in vitro* in complexes with CDKs [168]. The v-cyclin/CDK complexes are insensitive to CDK inhibitors such as p16INK4a, p21CIP1 and p27KIP1. Therefore, exogenous expression of v-cyclin from the infecting viral genome inhibits CDK inhibitors-imposed G1 arrest, and stimulates the cell cycle entry into S-phase [169]. Studies by Ojala *et al.* demonstrated that expression of v-cyclin in cells with elevated levels of CDK6 led to apoptosis after the cells entered into S-phase. Their results suggested that v-cyclin may employ both growth-promoting and apoptotic functions in KS, depending on factors regulating CDK6 and v-Bcl2 levels [170].

### 3.3. vBcl-2

ORF16-encoded vBcl-2 is 60% identical to the cellular Bcl-2, which has been shown to regulate apoptosis by dimerizing with other members of the family [171]. The vBcl-2 protein interacts with and inhibits the pro-apoptotic function of the cellular Bcl-2 family member, confirming that the cell survives for production of viral progeny. Several studies also suggest a novel role for viral BCL-2 members for preventing apoptosis [172]. In addition to apoptosis, autophagy plays a critical role in inhibiting intracellular pathogens and initiating CD4+ antigen presentation [173]. Interestingly, vBCL-2 inhibition of autophagy is considered an important mechanism for gammaherpesviruses to escape the innate immune response. Therefore, vBCL-2 may help KSHV to escape both apoptotic and autophagic surveillance systems [174]. Interestingly, vBCL-2 is expressed as an early gene during lytic replication [175], and apparently delays apoptosis to allow optimal virion production [176].

### 3.4. vIRFs

KSHV encodes vIRF (viral interferon regulatory factors of KSHV), which has significant sequence similarity to the human IRF family of proteins [177]. IFNs stimulate the anti-viral state in the target cell by modifying signal transduction pathways, such as increased major histocompatibility complex (MHC) I transcription, cell cycle regulation through induced transcription of p21 and possibly via p53 independent apoptosis [178]. KSHV-encoded IRFs share functional homology with human IRF-2, which is known to inhibit IFN-L signal transduction [179]. The function of IRFs is not restricted to the innate immune response, as they also play a major role in the modulation of cell growth, differentiation, and apoptosis. Thus, deregulation of these functions may lead to tumorigenesis [180]. Indeed, KSHV-vIRFs have been identified as effective inhibitors of interferon signaling and modulators of cellular oncogenic pathways [181]. Importantly, vIRF1 greatly reduces the levels of p53 phosphorylation on serine residue 15, resulting in an increase of p53 ubiquitination by MDM2, leading to p53 degradation [182]. Additionally, vIRF1 directly interacts with the DNA-binding domain (DBD) of p53 and suppresses p53 acetylation, resulting in inhibition of p53 activity and p53-mediated apoptosis [183]. vIRF1 was shown to suppress the TGF-β/Smad signaling pathway [184]. Moreover, vIRF1 and vIRF2 act as modulators of the immune system by repressing activation-induced cell death (AICD) via modulation of TCR/CD3-mediated induction of CD95L [185]. Interestingly, vIRF3 is required for the survival of KSHV-infected PEL cells *in vitro*, and silencing of vIRF-3 resulted in an increase of Caspase 3 or 7 activity and the subsequent induction of apoptosis [186]. In several studies on vIRF4, it has been shown to act as a potential antagonist of the Notch/CBF1 signal transduction pathway [181].

### 3.5. vGPCR

KSHV-G protein-coupled receptor (vGPCR or ORF74) is closely related to the IL-8 receptors CXCR1 and CXCR2 [169]. KSHV vGPCR has been demonstrated to activate the phosphoinositide pathway in COS-1 cells. Furthermore, *in vitro* transfection of rat fibroblasts with vGPCR leads to cell proliferation [187]. Studies showed that AIDS-associated KS expressed elevated levels of vascular endothelial growth factor which functions as an autocrine growth factor and is angiogenic [188], suggesting it could have a role in KS pathology. In addition, vGPCR expressing NIH3T3 cells has been shown to induce tumor formation after injection into nude mice [189]. In the context of KSHV life cycle, Bala Chandran’s group demonstrated a role for vGPCR in the sustained expression of ORF50, which could lead to a continued activation of lytic cycle genes and ultimately to successful viral progeny formation [190]. 

### 3.6. vMIP

KSHV-encoded chemokines or viral macrophage inflammatory proteins, including vMIP-I, vMIP-II and vMIP-III, show maximum similarity to cellular chemokines such as thymus activation-regulated chemokines, macrophage-derived chemokine and myeloid progenitor inhibitory factor-2 [191]. Interestingly, vMIP-I and vMIP-III were shown to be specific agonists for host CCR8 and CCR4, respectively, and it was demonstrated that Th2 T cells expressing CCR4 manifested an increased rate of migration towards vMIP-III in chemotaxis assays [192]. These viral-encoded macrophage inflammatory proteins have been demonstrated to increase angiogenesis in chick chorio-allantoic assays, suggesting a crucial role in the pathogenesis of KS [75].

### 3.7. vIL-6

The KSHV-encoded interleukin-6 or vIL-6 shares 24% identical amino acid sequence with human IL-6 [193] and cultured KS cells have been shown to respond to recombinant hIL-6, suggesting vIL-6 has a role in KS pathogenesis [168]. This proved that KS spindle cells express high affinity IL-6 receptor *in vivo* [193]. Wu *et al.* showed that vIL-6 promotes cell proliferation and migration by upregulating DNMT1 via STAT3 activation [194]. Studies by Giffin *et al.* indicated that hypoxia-upregulated protein 1 (HYOU1) is important for vIL-6 function and may play a role in the pathogenesis of KSHV-associated cancers [195].

### 3.8. vFLIP

FADD-like interleukin-1-beta-converting enzyme (FLICE/caspase-8)-inhibitory proteins or FLIPs were considered to bind adaptor proteins (TRADD and FADD) of the Fas/TNFR signaling pathway via their death effector domains (DEDs) [196]. This binding impairs the recruitment and activation of Caspase 8, leading to inhibition of the caspase activation cascade that results in apoptosis, and other herpesvirus encoded v-FLIPs have been shown to utilize this mechanism [196]. Some reports suggested that KSHV encoded v-FLIP can also function in this manner [197]. Other studies showed that KSHV v-FLIP can also block cell death via induction of the anti-apoptotic transcription factor NF-κB [198]. Interestingly, vFLIP can be detected in PEL cells complexed with NEMO [199]. Additionally, studies by An *et al.* demonstrated that vFLIP is responsible for a significant proportion of the constitutive NF-κB activity observed in PEL cells [200].

## 4. Role of Gammaherpesviruses in Targeting Signal Transduction Pathways

### 4.1. NF-kB Signaling Pathway

Several studies have indicated that the gammaherpesvirus life cycle can be regulated through the cellular nuclear factor (NF)-κB signaling pathway [201]. Expression of the NF-κB subunit p65 inhibits lytic replication of MHV-68, suggesting that high levels of NF-κB can promote establishment of latency [202]. In a resting non-activated state, NF-κB dimers are sequestered in the cytoplasm as a result of their association with the inhibitory protein IκBα [203]. On stimulation, phosphorylation of IκBα at serines 32 and 36 by IκB kinases induces ubiquitination and proteosome-mediated degradation [160]. Exclusion of the IκB protein exposes a nuclear localization sequence on the NF-κB complex resulting in translocation of the complex into the nucleus [204]. Therefore, NF-κB plays a critical role in multiple facets of MHV-68 pathogenesis. Regarding the role of CD40, one upstream activator of NF-κB has been examined in the context of an intact immune response. Studies by Blackman *et al.* examining infection of CD40+/CD40− mixed bone marrow chimeric mice reported that CD40-deficient B-cells latently infected with γHV68 rapidly waned compared to latency in CD40-sufficient B-cells [205]. This suggests that NF-κB activation upon CD40L stimulation plays an important role in the maintenance of latency in B-cells [205]. Replication of MHV-68 in cell culture is blocked by the over-expression of p65, and it has been suggested that NF-κB activation may promote the establishment of latency by inhibiting lytic cycle initiation [202]. Upon viral infection, the mitochondrial antiviral signaling (MAVS)-IKKβ pathway is activated to restrict viral replication. Manipulation of immune signaling events by pathogens has been considered as an outstanding theme of host-pathogen interaction. Studies by Pinghui Feng’s group showed that loss of MAVS or IKKβ impaired the lytic replication of gamma-herpesvirus 68 (γHV68), a model herpesvirus for human Kaposi’s sarcoma-associated herpesvirus and Epstein-Barr virus [206]. Additionally, Krug *et al.* examined how elimination of the NF-κB transcription factor p50 from mice affects the life cycle of murine gammaherpesvirus 68 (MHV68) [207]. Recently, Philip G. Stevenson’s group demonstrated that B cell proliferation driven by a murid γHV requires BAFF-R. This supports the idea that γHVs exploit host proliferation pathways and suggests that interfering with BAFF-R could more generally reduce γHV-associated B cell proliferation [208].

### 4.2. Wnt Signaling

Wnt signaling is considered to be important for embryogenesis and ontogenesis [209]. Wnt signaling is triggered by the binding of Wnt family ligand to their receptors, which are referred to as the Frizzled receptor family, and then transmitted to the Dishevelled protein, which inactivates glycogen synthase kinase-3β (GSK-3β) [210]. β-Catenin is generally phosphorylated by GSK-3β before undergoing degradation through the ubiquitin-proteasome pathway [211]. Wnt-induced inactivation of GSK-3β resulted in stabilization of β-catenin, causing its accumulation in the cytosol and subsequent transfer into the nucleus. In nucleus, it binds to the T-cell factor (TCF) family of transcription factors and induces the expression of Wnt-specific target genes which regulate cellular proliferation or differentiation [212]. Several in-depth studies suggest that EBV is capable of modulating Wnt signaling [213]. It has been suggested that LMP1 expression can repress the expression of E-cadherin [214]. A recent report showed that transient or stable expression of LMP1 sequences from normal B-cells and NPC does not impair the expression of E-Cadherin and other mediators of the Wnt pathway [170]. Furthermore, they also demonstrated that LMP1 expression in human cells had minimal effect on the interaction of E-cadherin and β-catenin and thus no evidence of β-catenin-mediated transcriptional activation [170]. Interestingly, LANA1 can act as a second pro-mitogenic factor through its ability to activate the Wnt-Signaling pathway [170]. Hayward and colleagues identified GSK-3β as a protein interacting partner with LANA1 [215]. GSK3-β normally phosphorylates β-catenin causing cytoplasmic sequestration of this proto-oncoprotein. LANA1, however, prevents GSK-3β regulation of β-catenin, allowing it to accumulate in the nucleus and transactivate responsive promoters, including the cMYC promoter. cMYC has various mitogenic activity and activates the hTERT promoter, which may contribute to the ability of LANA1 to activate telomerase activity in KSHV infected cell lines [216]. Inhibition of GSK3-β could potentially modulate cell-cycle entry through its ability to phosphorylate Cyclin-D, resulting in their cytoplasmic accumulation [217]. Interestingly, EBV has also been shown to stabilize β-catenin, showing conservation of cell signaling pathway targeting by these two viruses [218].

### 4.3. NOTCH Signaling

EBV-encoded EBNA2 and KSHV-encoded RTA are recruited to their responsive elements through association with the transcription factor RBP-Jκ [219]. RBP-Jκ binding sites are present in a number of EBNA2- and RTA-regulated viral promoters [172]. Biochemical and genetic analysis have established that RBP-Jκ acts downstream of the receptor Notch. Activation of the Notch receptor by binding of its ligand (Delta, Jagged, or Serrate) ultimately leads to the proteolytic cleavage of the receptor at the inner side of the membrane [220]. The Notch intracellular domain (NIC) is then translocated to the nucleus, where it activates genes by interacting with RBP-Jκ. EBNA2 and RTA may thus be regarded as functional homologs of the activated Notch protein. Interestingly, NIC has been shown to be capable of functionally replacing EBNA2 in the context of EBV infection for primary B-cell transformation [221].

Notch signaling has also been found to be linked to the pathogenesis of the other KSHV-associated malignancies including PEL [222]. The activated intracellular domain of Notch1 (NICD1) was shown to aberrantly accumulate in latently KSHV-infected PEL cells, resulting in increased proliferation [223]. Accumulation of NICD1 was observed to be dependent on LANA, which was shown to interact with an E3 ligase, SEL10 to mediate the ubiquitin-dependent degradation of NICD. LANA interrupts this ubiquitin-dependent degradation by competing with NICD for the association with SEL10 [224]. Growth of the KSHV-infected PEL cell lines such as BCBL-1, BC-3 and JSC-1 *in vitro* was dramatically arrested at the G1 phase by treatment with γ-secretase inhibitors. Furthermore, γ-secretase inhibitors treatment resulted in necrosis as well as apoptosis in tumors generated by the xeno-transplanted PEL cell lines. This experiment suggests that targeted downregulation of deregulated Notch signaling could have therapeutic potential for KSHV-related PELs [225].

### 4.4. P53 Signaling

As “guardian of the genome,” p53 acts as an internal sentinel for DNA damage response, cellular stress, including hypoxia, oncogene activation, starvation, altered mitochondrial and ribosomal biogenesis, denuded telomeres [226]. Depending on the level of cellular compromise, p53 can induce apoptosis, cell cycle arrest and the subsequent DNA repair mechanism. Deregulated p53 signaling contributes to cancer pathogenesis. Importantly, p53 is inactivated in 50% of human cancers, and components of the p53 signaling pathway, e.g., Mdm2 and p14Arf, are often misappropriated in the other 50% of cases. Additionally, molecular epidemiological analyses revealed that several cancers, including breast, head and neck, liver, and hematopoietic malignancies, showed a significant association of p53 mutations. Our lab demonstrated that EBV essential antigen EBNA3C substantially represses the transcriptional activity of p53 in luciferase based reporter assays, and rescues apoptosis induced by ectopic p53 expression in SAOS-2 (p53−/−) cells. Interestingly, the study also showed that the DNA-binding ability of p53 was diminished in the presence of EBNA3C (Figure 2) [136]. In other experimental systems, EBNA3C was found crucial for regulating p53 through induction of Aurora kinase B (Figure 2) [138]. Additionally, EBNA3C inhibits the function of p53 through the interaction with ING4 and ING5 (Figure 2) [135]. Interestingly, EBNA3C was observed to be important for Gemin3 stabilization to block p53-mediated apoptosis (Figure 2) [131]. 

**Figure 3:**
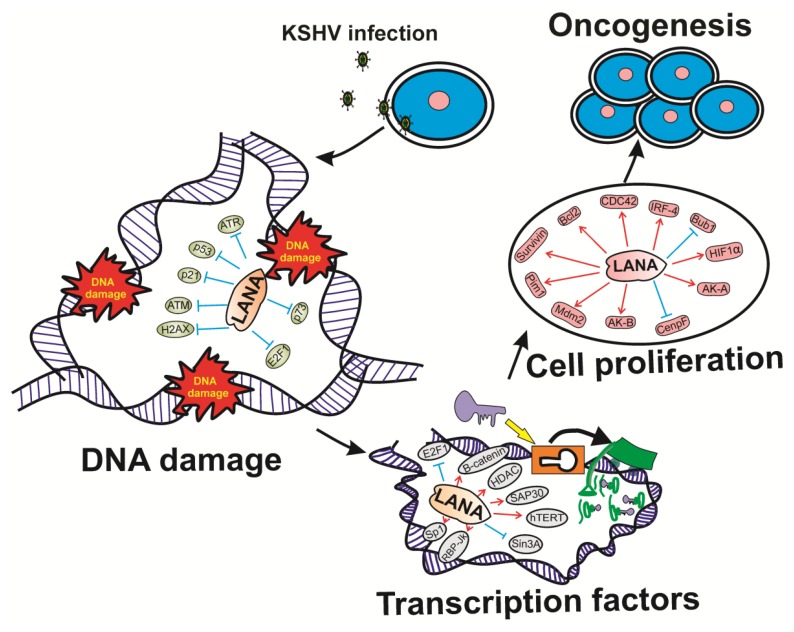
Schematic shows the vital role of KSHV-latency associated nuclear antigen (LANA)-mediated cancer progression by deregulating DNA damage response, transcription factor activities, and cell proliferation properties.

Previous works have suggested that ORF73 of herpesvirus saimiri, a viral homolog of Kaposi’s sarcoma-associated herpesvirus, modulates the two cellular tumor suppressor proteins p53 and pRb [227]. In the context of KSHV infection, latency-associated nuclear antigen (LANA) was found important in viral genome maintenance and interaction between LANA and the tumor suppressor p53 and pRb promoted oncogenesis (Figure 3) [158]. Importantly, cellular protein degradation pathways can be utilized by viruses to establish an environment that favors their propagation. Our lab showed that LANA can directly function as a component of the EC5S ubiquitin complex targeting the tumor suppressors von Hippel-Lindau (VHL) and p53 for degradation (Figure 3) [228]. In a more precise way, Cai *et al.* demonstrated that protein levels of Aurora A kinase is upregulated by LANA, and that elevated Aurora A level induces phosphorylation of p53, which enhances the interaction of LANA with p53, and promotes LANA-mediated p53 ubiquitylation and degradation, and hence inhibition of p53 transcriptional and apoptotic activities (Figure 3) [229].

### 4.5. MAPK Signaling

In contrast to EBV and KSHV, MHV68 provides a tractable small animal model for evaluating gammaherpesviruses pathogenesis that undergoes strong productive replication in cultured cells. In fact, MHV68 offers an attractive system for understanding the virus-host pathogenesis during productive viral replication [230]. Acute MHV68 infection was found necessary for viral dissemination and latency establishment in distal reservoirs [231]. While the importance of DDR, mitogen-activated protein kinase (MAPK), and inhibitor of kappa-B kinase (IKK) signaling in MHV68 replication was recently demonstrated [232], an understanding of how the kinases involved in these responses influence the overall phosphoprotein milieu within the host cell is not known. The breadth of host or viral proteins targeted by specific viral antigens with critical roles in pathogenesis, which include the conserved herpesvirus protein kinase (CHPK) ORF36 [233], or the viral cyclin D ortholog, encoded by gamma-2-herpesviruses such as KSHV [234], and MHV68 [235], also has not been evaluated. Arbiser *et al.* had demonstrated the synergy between inactivation of p16Ink4a and activation of mitogen-activated protein kinase (MAPK) [236]. Furthermore, they showed that cancers which are caused by reactive oxygen species (ROS) show high levels of hypermethylation of the tumor suppressor p16Ink4a and activation of MAPK pathway [237]. Recently, it was reported that the cellular activator protein-1 (AP-1) complex, formed by dimerization of c-Jun and c-Fos, activates early-lytic KSHV promoters, among them the Rta promoter in PEL cells (Figure 3) [238]. Recently, we demonstrated that EBNA3C encoded by EBV had differential expression patterns for the phosphorylation of MAP-Kinase when viral recombinants were used to infect primary B-cells [239]. This study strengthens the importance of MAP-Kinase in EBV-infected primary cells.

### 4.6. TLR Signaling

Toll-like receptors (TLRs) are considered fundamental components of the innate immune system. TLRs are categorized as pattern recognition receptors, as they are capable of recognizing invading pathogens [240]. 10 human TLRs have been identified to date. The TLR family of proteins are transmembrane proteins having immunoglobulin like extracellular domain and an intracellular domain which contains Toll/interleukin-1 (IL-1) receptor (TIR) domains through which the TLRs initiate their signaling [241]. All TLRs contain 21 to 25 leucine-rich repeats in their extracellular domains that recognize and bind to pathogen-associated molecular patterns on the surface of the incoming pathogen [242]. However, all TLRs are not expressed at the cell surface. TLR3, 7, 8, and 9 are all expressed in intracellular compartments [243]. Recently, TLR2, 3, 4, 7, 8, and 9 were identified as being involved in the recognition of viruses through binding to RNA, DNA, or viral glycoproteins [243]. Additionally, TLR3 and TLR7 have been reported to be induced by RNA. TLR3 can recognize single-stranded and double-stranded RNA, while TLR7 can recognize only single-stranded RNA [240]. Recent evidence implicated a role for TLRs in herpesvirus infection. TLR2 and TLR4 have been shown to recognize several herpesviruses, including herpes simplex virus 1 (HSV-1) and human cytomegalovirus [244]. Epstein-Barr virus (EBV) has recently been shown to upregulate TLR7 [245]. Additionally, Zhang *et al.* have recently reported that TLR3 deficiency in humans can lead to the uncontrolled spread of HSV-1 and HSV-1-associated encephalitis [246]. Additionally, another study demonstrated that TLR stimulation can drive MHV68 reactivation from latency and suggests that periodic pathogen exposure may contribute to the homeostatic maintenance of chronic gammaherpesvirus infection through stimulating virus reactivation and reseeding latency reservoirs [247]. Studies by Blossom Damania’s group showed that agonists specific for TLR7/8 reactivated latent KSHV and induced viral lytic gene transcription and replication. Furthermore, vesicular stomatitis virus (VSV), a bonafide physiological activator of TLR7/8, also reactivated KSHV from latency [248].

## 5. Gammaherpesviruses Deregulation of Cellular Processes: Deregulation of Apoptosis

The intrinsic, or mitochondrial-mediated apoptosis pathway is governed by B-cell leukemia/lymphoma 2 (Bcl2) family of proteins [249]. This important family includes both pro- and anti-apoptotic proteins, which share homology to the conserved Bcl2 homology (BH) regions [250]. It is well-known that KSHV and EBV express homologs of Bcl2; however, a detailed mechanism by which these proteins function remains somewhat elusive [251]. Various studies demonstrated that heterologous expression of these proteins resulted in resistance to apoptosis from numerous pathways [252]. However, interactions with pro-death Bcl2 family members could not be clearly demonstrated. Earlier it was suggested that KSHV-encoded Bcl2 does not interact with BAK, BAX, BAD or BIK. However, another group showed that it can interact with BAK but not BAX (Figure 3) [253].

Apoptosis is required for embryonic development, organogenesis and the removal of damaged or aged cells during the maintenance of cellular homeostasis [252]. Under physiological condition, apoptosis is strictly regulated. When this regulation fails, a number of pathologies may result, such as autoimmune or neurodegenerative diseases and cancer [252]. During apoptosis the chromatin is condensed and fragmented, forming vesicles of different sizes which is surrounded by a plasma membrane [254]. These vesicles, identified as apoptotic bodies, contain chromatin and cellular organelles [255]. Our study using AK-B knockdown leads to similar phenomenon where we observed nuclear blebbing by changes in nuclear shape from blebs to spikes to blisters (Figure 2) [137]. The final phase is the termination phase, which involves phagocytosis and degradation of the apoptotic bodies [256]. Apoptotic death can be triggered by different intra- or extracellular stimuli. We showed that in the presence of EBV-encoded EBNA3C, knockdown of AK-B no longer induces apoptosis [137]. We also demonstrated enhanced levels of Caspases 3 and 9 after the knockdown of AK-B or Pim1 in lymphoblastoid cells (Figure 2) [137]. Interestingly, p53, a critical tumor suppressor in EBV associated lymphoma, and p73 activities are deregulated when AK-B as well as EBNA3C are expressed (Figure 2) [138]. In apoptosis, the intrinsic pathway is regulated by mitochondrial proteins that, upon activation, cause the release of cytochrome C into the cytoplasm. In the cytosol, a complex known as the Apoptosome is formed through the binding of Apoptotic Protease Activation Factor 1 (Apaf-1), Procaspase 9, and Cytochrome C (Figure 2) [137]. The oligomerization of Apaf-1 activates Caspase 9, which, in turn, induces the proteolytic cleavage of other substrates involved in cell death (Figure 2) [137]. The Caspases constitutes a family of cysteine proteases that are specific for aspartate. The Caspase family members are similar in amino acid sequence, structure, and specificity [137]. Caspases are synthesized as zymogens, and their activation requires specific cleavage at selected aspartate residues [257]. At the initial processing, an inactive caspase is cleaved in large (p20) and small (p10) subunits, after which the N-terminal domain is removed to form the catalytically active protease [258].

The EBV-encoded latent antigens EBNA1, EBNA3C and the viral miRNAs are shown to be involved in BL/lymphoblastoid cell proliferation and/or resistance to apoptosis, thus conferring a selective advantage to neoplastic cells [259]. Recently, we observed that the DNA damage response molecule H2AX which also inhibits cell proliferation is also targeted by EBNA3C during EBV infection (Figure 2) [260]. We also demonstrated that knockdown of H2AX in LCLs led to activation of mitotic protein Bub1 and reduction of tumor suppressor p53 (Figure 2) [260]. Additionally, LMP1 was shown to mimic CD40 induced signaling by providing erroneous survival signals in viral infected B-cells within the germinal center [261]. LMP1 contributes to neoplastic transformation and tumor progression by modulating the TNF receptor pathway through its interaction with CTAR1 and CTAR2 domains in a ligand-independent manner [262]. In turn, these domains interacted with factors associated with TNF-R (TRAFs) and death domains coupled with TNF-R (TRADDs) [263]. The association of LMP1 with the TRAF and TRADD molecules activates a signaling cascade that results in the constitutive activation of the JNK, NF-κB and PI3K pathways [264]. Numerous studies from our group and others showed that EBNA3C can also play a role in apoptosis control [137,239,260]. Moreover, a Bac-recombinant EBV deleted for a specific region of EBNA3C also supported our initial studies [239].

KSHV oncogenic activity is supported by the numerous pro-angiogenic molecules that are induced following infection of endothelial cells, including the VEGF-VEGFR family, cyclooxygenase 2 (COX2) and angiogenin [265]. However, in the general population, KSHV infection rarely leads to KS, indicating the need for additional cofactors including immunosuppression that leads to tumor induction [266]. LANA is one of the encoded oncoproteins due to its ability to deregulate tumor suppressor pathways associated with p53 and pRb and to transform primary rat embryo fibroblasts and mouse embryonic fibroblast in co-operation with the cellular oncogene H-ras [158]. Additionally, LANA also deregulates Wnt signaling by altering the subcellular distribution of glycogen synthase kinase 3 (GSK-3) (Figure 3) [267]. LANA modulates apoptosis by direct binding to p53 and H2AX (Figure 3) [160,268]. The encoded vCyclin (viral homolog of cellular cyclin D) is a constitutive activator of cyclin dependent kinase 6 (CDK6). The expression of vCyclin and the formation of the complex vCyclin/CDK6 leads to defects in cytokinesis, which results in polyploidy and the activation of p53 [269]. Furthermore, vIRF1 inhibits p53-induced apoptosis through its interaction with the central DNA-binding domain of p53 and with the upstream ATM kinase [182]. The K1 membrane protein, which is the first ORF of KSHV, inhibits apoptosis by inducing the release of the growth factors VEGF, leading to the subsequent activation of the PI3K-AKT pathway [253]. Prior to cell lysis, the inhibition of apoptosis by lytic proteins could also contribute to cell transformation, viral replication and virions production and assembly [270].

Caspase-3 is a downstream effector of both receptor-dependent and receptor-independent apoptotic stimuli, and its activation has served as a marker for cells undergoing apoptosis [270]. To investigate a role for K1 in protection of KSHV-positive B-cells from Fas-mediated apoptosis, one study showed that the expression of K1 in BCBL-1 cells resulted in a 40% decrease in Caspase-3 activity induced by engagement of the Fas receptor, compared to BcL2 that resulted in a 60% decrease in Caspase-3 activity [270]. Hinshaw *et al.* showed that this protection is mediated upstream of caspase activation [271]. While K1 protected cells from FKHR-induced apoptosis, it did so to a lesser extent when compared to that seen with Fas-mediated apoptosis [270]. K1 can also activate the Akt pathway in B-lymphocytes and that this activation is mediated by PI3K [272]. This is consistent with other reports demonstrating that Akt is a target for other transforming viral gene products, namely simian virus 40 large and small T antigens, and EBV-LMP1 and LMP2A proteins [273]. Similar to K1, the KSHV vGPCR protein has also been shown to transform cells and to target Akt kinase [274].

## 6. Modulation of Autophagy

Autophagy is a series of catabolic processes required for quality control of proteins and organelles of eukaryotic cells. Simultaneously, this process also emerged as an essential part of the host antiviral defense [275]. Recently, it has been suggested that cellular autophagy can significantly affect viral persistence and may also influence the *in vivo* fitness of viruses [276]. The autophagy signaling pathway controls varied cellular growth, differentiation, and homeostatic processes [277]. It also involves lysosome dependent bulk degradation of cytosolic proteins and organelles. It sustains cellular homeostasis as well as defends cells from assaults by many pathogens. Moreover, dispensation of autophagy delivers signaling for viral recognition, IFN secretion (Th-1), as well as endogenous MHC class II antigen presentation [278].

Previous studies suggested that vBcl2 induces caspases independent autophagic cell death and antagonizes the Beclin1-mediated autophagic death of chronically infected cell death [279]. It also suggested that the physiological role of vBcl2 is not only limited to the inhibition of Fas-mediated apoptosis. Inhibition of Beclin1-mediated autophagy by vBcl2 has opened an alternative survival signal for persistent amplification of virus [280]. Moreover, Tang *et al.* suggested that vBcl2 can prevent apoptosis [281]. In normal physiological conditions, basal autophagy has a housekeeping function, allowing cells to recycle organelles and long-lived proteins. However, autophagy is also induced in response to various forms of stress, including starvation, depletion of growth factors, low energy levels, hypoxia, oxidative stress, ER stress and pathogenic infection [282]. Autophagy has also recently been shown to be involved in diverse aspects of the innate and adaptive immune response [283].

The mTOR kinase is essential for controlling the autophagic process by growth factors, insulin, nutrients, calcium signaling, ATP level, hypoxia and oxidative stress [284]. The key inhibitor of autophagy is present in two different complexes, including mTORC1 and mTORC2, where it is associated with Raptor and Rictor, respectively [285]. The mTORC1 complex controls protein synthesis, the importation of nutrients and autophagy [286]. Inhibition of mTORC1 by Rapamycin activates autophagy in all eukaryotic cells. The downstream targets of mTORC1 that regulate autophagy in mammalian cells are ULK1 and ULK2 [287]. Autophagy is known to be upregulated in stress induced eIF2, kinase signaling pathway, whereas the mechanism is still unknown [288]. PKR is induced by interferon and is activated by double stranded RNA, a common intermediate in the replication of many viruses [289]. PKR is a key player in the antiviral action of interferon and many viruses express proteins that antagonize the PKR signaling [290]. In herpesvirus-mediated pathogenesis, autophagy plays a role in antiviral cell defense either by clearing intracellular viruses or by activating antigen presentation [291]. Therefore, it is hypothesized that herpesvirus evolved intricate mechanisms to antagonize autophagy. Studies suggested that a number of herpesvirus proteins have been reported to target the autophagy machinery [286]. However, a few cases also reported cases where herpesviruses are not able to inhibit autophagy [292]. Fewer studies were conducted on autophagy and lytic cycle of EBV, while two latent antigens, EBNA1 and LMP1, are known to activate or to interfere with the autophagic machinery [293]. As endogenous antigens, EBNA1 would be expected to load onto MHC class I after proteasomal processing [294]. However, EBNA1 elicits a CD4+ T cell immune response after intracellular MHC class II processing [224]. Earlier, Paludan *et al.* showed that autophagy contributes to MHC class II-EBNA1 presentation in EBV transformed lymphoblastoid cells [293]. Moreover, EBNA1 delivered to the lysosomal compartment, and at the same time inhibition of lysosomal acidification induces an accumulation of EBNA1 in autophagosomes in Hodgkin’s lymphoma cells [293], whereas suppression of autophagy by 3-methyladenine or by small interfering RNA restrained the intracellular processing of EBNA1 and consequently its MHC class II presentation [286]. The induction of autophagy is also associated with LMP1 expression; low level LMP1 expressing cells displayed autophagosomes, while cells with enhanced LMP1 expression displayed autolysosomes [286]. In addition to this, transmembrane domains 3–6 of LMP1 are responsible for activating autophagy [295].

Autophagy is considered a well-established cell survival mechanism. There is also evidence of cross-talk between autophagy and apoptosis [296], as several mediators of apoptosis have been shown to control autophagy. Therefore, it would not be impossible to find that some anti-apoptotic proteins can also inhibit autophagy. Interestingly, vBcL-2 homologs, vBcl-2 and BHRF1 have been reported in HHV-8, and EBV, respectively [297].

## 7. Epigenetic Regulation by Gammaherpesviruses

Epigenetic mechanisms are fundamental to the regulation of many different cellular processes like gene expression, DNA-protein interactions, and suppression of transposable element mobility, cellular differentiation, embryogenesis, X-chromosome inactivation and genomic imprinting [298]. Deregulation of epigenetic processes is associated with several diseases such as cancer, neurodevelopmental disorders, neurodegenerative and neurological diseases and autoimmune diseases [299]. The silencing capability of CpG methylation is thought to be due to the impaired binding capability of transcriptional activators to methylated DNA and the increased binding affinity of transcriptional repressors [300]. Additionally, inhibition of transcription is accomplished by permitting the binding of methyl-CpG binding domain (MBD) proteins such as MDBP-1, which can then block access of transcription factors to DNA [301]. In mammalian cells *de novo* methylation is performed by DNA methyltransferase 3 (DNMT3A and DNMT3B) [302]. Methylation patterns are transmitted by mitotic inheritance via members of the maintenance methyltransferase family DNMT1 [303]. Besides DNA methylation, post-translational modification (PTM) of core histone proteins is a key factor in epigenetic regulation. The nuclear DNA is wrapped around histone octamers that consist of two copies of each H2A, H2B, H3 and H4, thereby forming a nucleosome structure [304]. Core histones are predominantly globular, except for their N-terminal tails, which are unstructured and accessible for PTM. These modifications primarily occur at lysine residues and include acetylation, methylation, phosphorylation, ubiquitylation and SUMOylation [305]. They have been shown to play important roles in the regulation of transcription, DNA damage response, DNA replication, alternative splicing, nuclear organization and chromosome condensation [306]. In general, chromatin is roughly divided into transcriptionally inactive heterochromatin and actively transcribed euchromatin; however, several intermediate states have been described. Among many potential histone modifications associated with the chromatin state, a few have been extensively studied and now represent widely accepted markers for the respective chromatin state [273]. Euchromatin is characterized by high levels of acetylation of histone 3 lysines 9 and 14 (H3K9/K14-ac) and tri-methylation of H3K4, H3K36 and H3K79, whereas heterochromatin is found to carry low levels of acetylation marks but high levels of tri-methylated H3K9, H3K27 and H4K20 (Table 1). In general, the different modifications provide binding sites for factors and complexes that lead to the formation of either densely packaged heterochromatin or open and transcriptionally accessible euchromatin. The list of histone modifying enzymes and those who are capable of removing them is long and indicates that post-translational histone modification at least in part represents a highly dynamic process. Dhiab *et al.* demonstrated that epigenetic alterations are common in both EBV-positive and EBV-negative Hodgkin’s Lymphomas (HLs), and that the frequencies of changes varies according to several clinico-pathological parameters, such as age and sex. The latter likely reflects the multitude of environmental factors involved in the pathogenesis of HL and the complexity of their interactions with genetic and/or hormonal factors [307].

**Table 1:** Epigenetic markers: activation and repression.

Activation Markers	Repression Markers
H3K4me3	H3K27me3
H3K36me3	H3K9me3
H3K79me3	H3K20me3
H3K9Ac	
H3K36Ac	
H3K4me3	
H3K36-me2/3	
H3K79-me2/3	

### 7.1. Role for H3 Lysine 27 Trimethylation

Recent studies indicate a role for H3 lysine 27 tri-methylation (H3K27) in viral-mediated tumorigenesis [308]. In EBV infection, it has been shown that histone H3K27me3 and H4K20me3 markers are crucial for suppression of BZLF1 in latent Raji cells [309]. Additionally, H3K9me2/3, heterochromatin protein 1, and H2A ubiquitination are associated with latency, while positive histone markers, such as higher histone acetylation and H3K4me3, are associated with reactivation [309]. Transitions and differences in EBV infection cycling between lytic and latent states are going simultaneously, not only with the virus production and spread, but also with disease progression and malignancy of EBV-associated cancers [309]. We monitored the methylation status of tumor suppressors during EBV infection of PBMCs in a temporal manner [310]. In this study, we monitored the deregulation of tumor suppressors and cell-cycle checkpoints at both G1/S and G2/M transitions in EBV infected B-cells. Interestingly, this mechanism occurs through alteration of CpG methylation. Therefore, we suggest that CpG methylation of TSGs could be used as prognostic markers, as well as development of potential therapeutic avenues in EBV-associated cancers [310].

Chen *et al.* suggested that a single CTCF binding site controls LMP2A and LMP1 promoter selection, chromatin boundary function and episome copy number control during EBV latency [311]. They also hypothesized that CTCF166 is critical for maintaining a euchromatin histone modification pattern at the promoter regions of LMP2A and LMP1. Moreover, CTCF166 deleted at this region resulted in an increased heterochromatic H3K9me3 and CpG DNA methylation.

The importance of histone H3K27me3 in the maintenance of latency was demonstrated for KSHV ORF50/K-Rta [312]. In our study on PBMCs infected KSHV, we had shown that histone modifications across the KSHV genome presented a unique pattern of histone modifications, specifically with H3K4me3, H3K9me3, H3K27me3, and H3Ac in coarse of early infection [280]. Exclusively, we showed that histone modifications were exhibited in a temporal manner, suggesting that KSHV early infection is a dynamic process. Additionally, most of the epigenetic modifications were suppressed at 7 dpi. These findings suggested that early epigenetic modifications were essential to the successful establishment of latent infection in B cells post-infection of PBMCs. Studies also showed the involvement of Ezh2 methyltransferases and of the histone H3K27me3 marker in the silencing of BZLF1 gene expression during EBV latency [313]. Interestingly, another constitutive heterochromatin marker histone H3K9me3 was also reported to definitely be present during latency in EBV Zp and KSHV ORF50/K-Rta promoters [314]. Arvey *et al.* showed the CTCF binding downstream of this promoter and appears to serve as a boundary for high-level promoter proximal histone modifications H3K27ac and H3K4me3 [315].

### 7.2. Hyperacetylation of Promoters

In KSHV infection, hyperacetylation of histones take places primarily on promoters and associated with gene activation, while hypoacetylation is characteristic of repressed genes [316]. Histone methylation is associated with either activation or repression of genes, depending on which histone lysine residues are mono-, di- or trimethylated. Usually, transcriptionally active genes are correlated with H3K4me3 and H3K36me3, whereas tri-methylation of H3K9, H3K27 and H4K20 occurs mainly on repressed genes (Table 1) [317]. H3K9me3 and H4K20me3 histone modifications are characteristic of pericentric heterochromatin [318]. H3K27me3 is a marker for highly dynamic and reversible heterochromatin, and specifically represents genes that are subject to tissue specific or developmentally regulated expression [319]. An earlier study revealed that genome-wide analysis of embryonic stem (ES) cells for H3K27me3 is mainly situated on developmental genes [320]. Moreover, our study on primary cells infected with KSHV revealed that H3K27me3 modifications were widespread and efficiently occurred on the viral genome, and also included gene functions associated with anti-apoptosis, DNA replication, oncogenesis, immunomodulation, regulation of transmembrane protein, and virion particle assembly [321]. Importantly, the promoters of large numbers of these developmental genes are also enhanced in activating H3K4me3, indicating that these genes are silenced but poised for rapid activation in ES cells [322]. Primary cells infected KSHV demonstrated that H3K4me3 activity is associated with genes related to anti-apoptosis, DNA replication, oncogenesis, and virion particle assembly [321]. Interestingly, promoters enriched in both activating (H3K4me3) and repressive (H3K27me3) histone marks, called bivalent promoters, have been associated with rapidly inducible genes in T-cells as well PBMCs [323].

KSHV genes are correlated with a characteristic pattern of active and repressive histone modifications during latency, which eventually changes upon reactivation [312]. Importantly, the promoter regions of RTA and several early lytic genes are associated with both H3K4me3 and H3K27me3 marks, demonstrating that these crucial promoters have a bivalent chromatin structure that sustains their repression during latency [324]. This process eventually results in diminished H3K27me3, which are associated with increasing levels of activating histone marks on the RTA promoter [325]. Furthermore, treatment of KSHV latently infected cells with a drug inhibiting the expression of PcG proteins, the small inhibitory RNA mediated knockdown of EZH2, or over-expression of H3K27me3 histone demethylases efficiently triggers lytic reactivation of KSHV [312]. Moreover, PcG proteins are involved in the maintenance of KSHV latency by preserving an opposite heterochromatin on the promoter regions [312,326].

The EBV genome is found to be associated with frequently spaced nucleosomes during latency, similar to bulk cellular DNA [327]. Both cellular and viral chromatin can be organized into domains characterized by distinct histone modifications [328]. Chromatins with active transcription are typically characterized by high acetylation of histones H3 and H4 and high methylation of lysine 4 on histone H3 [328]. Additionally, those chromatins non-permissive for transcription are typically characterized by low acetylation of histones H3 and H4 and high methylation of histone H3 and K9 [329]. Previous studies have indicated that the EBV latency control region (LCR) is enriched in histone H3 methyl K4 and that the expansion of this modification to the Cp from OriP correlates with Cp activation in EBV infection [330]. Dynamic changes in chromatin organization may be mediated by insulator elements and chromatin boundary factors [331]. The cellular factor CTCF, or CCCTC-binding factor, is a central regulator of chromatin boundary elements [332]. CTCF was originally found as a repressor of the c-Myc but was later found to be involved in enhancer blocking, chromatin insulation, and imprinting on diverse genes [333]. The H3meK27 modification has been linked to transcription repression through polycomb-associated proteins [334]. One study also observed low levels of H3meK27, with a small peak for Raji cells between OriP and Cp [301]. H3meK36 has been associated with RNA polymerase II activity and transcription elongation [335]. H3mK36 was shown to be highly elevated in the cellular genes for actin, MTA1, and CD30 [303]. However, relatively low levels of H3meK36 were detected in the EBV genomes [336]. The Cp promoter can be silenced epigenetically by cytosine methylation during the transition from type III to type I latency [337]. However, factors that determine the sites of CpG methylation and the effectors of cytosine methylation-directed transcription repression are not completely understood [337]. H3meK4 is enriched at the OriP region in type I and type III latency, whereas H3meK4 may be most highly enriched at the EBERs, and RNA polymerase III transcription may be the triggering event for this histone modification [329]. H3meK4 extends through the OriP region in all cell types and is generally spread over a larger domain in type III latency [338]. However, H3meK4 modification may not be distributed uniformly throughout the regions between OriP and Cp. In lymphoblastoid cells, H3meK4 and H3meK9 appear to vacillate, with some partial overlap in the regions between the EBERs and Qp. These modifications are conserved through the replication of virus as well [163], as our earlier report showed that Survivin phosphorylation on residue T34 upregulates the activities of histone acetyl-transferases and deacetylases, which ultimately leads to an increased viral copy number in KSHV-infected B cells [163].

### 7.3. Deposition of H3me3K27

H3me3K27 is deposited by the evolutionary conserved 600-kDa Polycomb Repressive Complex 2 (PRC2), which consists of three PcG proteins (EZH2, SUZ12, EED) and the histone-binding proteins, RbAp48/46 [339]. The SET domain containing EZH2 is an H3me3K27 histone methyltransferase, which can be found along the entire genomic regions enriched with H3me3K27 in mammalian cells [340]. H3me3K27 provides a binding platform for PRC1, a larger Polycomb complex consisting of more than 10 subunits [341]. Polycomb-mediated gene silencing is reversible with H3me3K27 demethylases, such as JMJD3 and UTX, which can be recruited to the repressed promoters by transcription activators as was shown, for instance, in the case of the H3me3K4 methyltransferase complexes [342].

The enzymes modifying histone acetylation are known as acetyl-transferases (hKATs or HATs) and histone deacetylases (HDACs) [343]. It is important to note that H3K9/K14-ac and H3me3K4 modifications reflect transcription initiation and are not directly linked to elongation, which has been recently associated with di- and tri-methylation of lysines 36 and 79 of histone H3 (H3K36-me2/3 and H3K79-me2/3) [344]. Therefore, the presence of histone modifying enzyme-complexes and their differential influence on cellular transcription, represent a more dynamic process than DNA methylation [345]. Thus, we assumed that histone PTM may be present at early time points after *de novo* infection when the cells have already adopted a latent expression profile but fail to show DNA methylation. The following studies were performed with SLK cells infected for 5 days with KSHV (SLK5-dpi) and SLKP cells, which represent a long-term *de novo* infected population [346]. This would determine whether emerging modifications are general hallmarks of latent KSHV genomes.

In contrast to H3me3K9, the EZH2-mediated modification of histone H3me3K27 was found to cover large parts of the KSHV episome in both BCBL1 and SLKP cells which further exhibited a high similarity to their obtained distribution profiles [347]. Additionally, H3me3K9-occupied regions mentioned above were to some extent decreased in tri-methylated H3me3K27 levels in BCBL1 [348]. Furthermore, when compared to activating modification profiles, several regions exhibited hallmarks of bivalent chromatin, *i.e.* the simultaneous presence of activating (H3K4-me3 /H3K9K14ac) and repressive (H3K27me3) histone modifications [349]. Interestingly, the promoter region of the lytic cycle inducer Rta (ORF50) was featured prominently among these bivalent regions [347]. Therefore, gene silencing through polycomb-mediated tri-methylation of H3me3K27 may represent a key event in latency establishment upon *de novo* infection [350]. This hypothesis was further substantiated by retroviral expression of the H3me3K27 specific demethylase JMJD3, which was found to impair the stability of latency [351].

### 7.4. Methylation and Latency

It may well be that EBV and KSHV follow different strategies to achieve gene silencing during latent infection as in the case of DNA methylation, as it has been shown that EBV (in contrast to KSHV) becomes DNA methylated within a few days upon *de novo* infection, but following latency establishment, and that this methylation is a pre-requisite for efficient completion of the viral life cycle [352].

In different studies, DNA methylation displayed a strictly negative correlation to activating histone marks and was predominantly present in regions enriched in repressive H3K27me3 [341]. The issue arises whether histone modifications influence the delayed onset of profound DNA methylation and whether this interaction plays a role at later stages of viral latency. It seems likely that DNA methylation patterns were established as a consequence of the continuous presence of PRC2 on viral DNA, as such repressor complexes have been shown to recruit DNA methyltransferases either directly or indirectly via recruitment of PRC1 [353]. All possibilities likely contribute synergistically to the onset of late DNA methylation. It is very conceivable that DNA methylation may represent an additional, functionally important block which augments repressive histone marks and reinforces latent expression patterns during long-term latency *in vivo*, but which may be of lesser consequence in cell culture models of viral infection [347].

When Cp DNA is methylated, transcription initiates at Qp to generate a smaller transcript that produces EBNA1, but none of the other EBNA genes [354]. This switch from Cp to Qp is thought to occur during natural infection as B-cells differentiate from proliferating centroblasts to resting memory B-cells [355]. Concurrently, methylation occurs at the promoter regions for LMP1 and LMP2, which result in their stable repression. Notably, in BL, Qp is the default promoter for EBNA1 in type I latency, and OriP is the episome maintenance element [356]. Since EBNA1 binds to both of these regions, it has been proposed that EBNA1 prevents local CpG methylation [357]. Viral-encoded factors can alter the DNA methylation machinery by targeting DNA methyltransferases, DNA methyl binding proteins (MBPs), MBP-associated co-repressor complexes, and direct binding to methylated DNA [358]. KSHV LANA binds to the *de novo* methyltransferase DNMT3a [359], and the methyl cytosine binding protein MeCP2 [360]. In KSHV infection, LANA can stabilize the repression of KSHV lytic cycle gene expression, and its interactions with the DNA methylation machinery may partly account for this repressive activity. Consistent with this finding is the observation that DNMT3a and 3b mediate CpG methylation and transcription repression of MHV68 ORF50 promoter in latently infected B-lymphocytes *in vivo* [361]. The EBV lytic activator Zta has the unusual capacity to bind selectively to methylated DNA, enabling the establishment and reactivation of methylated genomes [362]. Viral DNA methylation patterns can also be regulated by noncoding RNAs [363]. Viral genomes appear to utilize cellular transcription factors and chromatin regulators indistinguishably from host chromosomes [354]. Most of the viral promoters share conventional features of cellular epigenetic regulation, with histone acetylation and H3K4me3 methylation occurring at transcribed promoters [364]. Increased histone H3 and H4 acetylation has been demonstrated for the activated Cp in response to EBNA2 activation in type III latency [365], and for activation of the EBV BZLF1/Zta [366] and KSHV ORF50/Rta immediate early gene promoters [367]. However, it remains possible that viral episomes are distinguished from cellular chromosomes by some as yet unknown mechanism. Examination of the EBV epigenome revealed sharp peaks of histone H3K4me3 and acetylated histones (H3K27ac and H3K9ac) at sites of transcription initiation for EBERs, Cp, LMP1/LMP2, and RPMS1p [315]. Therefore, the EBV epigenome showed clustering of multiple transcription factors at sites of active promoters, and very few interactions at transcriptionally silent genomic locations [368].

## 8. Conclusions

The molecular mechanisms behind EBV- and KSHV-mediated cell transformation continue to be investigated. Anti-viral drugs for KSHV-derived diseases in humans are essential for treating patients with advanced AIDS. Earlier reports suggested that GCV treatment caused rapid and dramatic declines in the prevalence of new KS tumors [369]. Exploration to identify new treatments against KSHV-associated malignancies is associated with identification of several genes and their products that directly contributed to associated malignancy [370]. Recently, a novel role for Ser/Thr Aurora Kinase, Pim kinases and replication genes in EBV and KSHV pathobiology has been reported. These kinases may be important targets for inhibiting lymphoblastoid and PEL tumor development [129,137,138,163,239]. This could be feasible to selectively inhibit these kinases to prevent or reduce resistance of PEL or lymphoblastoid cancers to specific treatments. Moreover, many cancers are targeted using Aurora kinase and Pim1 inhibitors for reducing tumor burden. A prognostic approach where using small molecules inhibitors targeting key signaling pathways in these viral infected cells may be useful in combination with kinase inhibitors [371]. Moreover, *in vivo* studies will be critical for validation of these strategies followed by clinical trials. Whether the establishment of virus latency is considered to be beneficial or unfavorable, both views aspire to attain the common goal for improving human health and alleviating human suffering from virus-associated diseases through either maintenance of latency or the development of vaccines. As compared to other viruses, development of herpesvirus antivirals is at the forefront of current research [163]. The presence of latent virus in EBV- and KSHV-associated cancers provides a potential target for therapy. Since currently available antiviral drugs target lytic replication but are ineffective against the virus in the latent state, drugs that reactivate the virus have been investigated as therapies for virus-positive neoplasia [372]. Therefore, in-depth understanding of both mechanisms of latency establishment and lytic reactivation would be an important strategy for achieving potential antiviral therapeutics, especially in the context of gammaherpesvirus-associated malignancies.
